# Predictive Factors for Total Knee Arthroplasty: An Observational Study

**DOI:** 10.7759/cureus.67519

**Published:** 2024-08-22

**Authors:** Sergiu Iordache, Adrian Cursaru, Bogdan Cretu, Cosmin-Florentin Niculae, Mihnea Popa, Mihai Aurel Costache, Bogdan Serban, Catalin Cirstoiu

**Affiliations:** 1 Orthopaedics and Traumatology, Bucharest Emergency University Hospital, Bucharest, ROU; 2 Molecular Pathology, Bucharest Emergency University Hospital, Bucharest, ROU

**Keywords:** injury risk, predictive factor, joint preservation, arthrosis, knee arthroplasty

## Abstract

Introduction

Knee osteoarthritis (KOA) is the most common form of osteoarthritis. It is diagnosed based on clinical symptoms, physical examination, and imaging, most frequently by knee X-ray in at least two views.

In front of a patient with early KOA, all the predictive factors and risk factors that can be modified, but also that can lead to a rapid evolution of the symptoms and the need for total knee arthroplasty (TKA), must be taken into account and identified. There were a series of prognostic factors associated with KOA, such as age, sex, BMI, degree of physical activity, decrease in bone mineral density, C-reactive protein, malalignment, clinical severity at baseline, and previous traumas.

The treatment of KOA is varied and involves pharmacological and non-pharmacological measures and surgical treatment in the final stages of evolution.

Materials and methods

In the University Emergency Hospital of Bucharest, Bucharest, Romania, patients with KOA presented to the Department of Orthopedics and Traumatology, and those who required surgical treatment, such as TKA, arthroscopy, or non-surgical treatment, were enrolled. We conducted a descriptive and prospective observational study that included 70 patients clinically and imaging diagnosed with KOA. The inclusion criteria consisted of a patient over 45 years old with knee pain and radiological signs of KOA. The exclusion criteria referred to patients with recent traumatic history, signs of active osteoarticular infection, the lack of radiological changes of KOA that imposed the differential diagnosis, patients known to have rheumatological diseases in the Algic phase, and patients in whom it was not possible to collect complete required data.

Results

Patients who required TKA were older (65.12 ± 8.19 years) than patients who required other therapeutic interventions (52.55 ± 3.63 years), the difference of 12.57 years being statistically significant (t = −8.882, p ≤ 0.001). Women were more than three times more likely to require TKA than men (80.85% vs. 52.17%, OR = 3.87, CI [1.29, 11.56]). Patients with HBP were more than four times more likely to require TKA than patients without HBP (78.57% vs. 42.86%, OR = 4.88, CI [1.42, 16.82]). Patients with elevated ESR were more than 26 times more likely to require TKA than patients with normal ESR (96.67% vs. 52.50%, OR = 26.23, CI [3.25, 211.67]). There are statistically significant differences between the non-TKA group (mean rank = 60.20) and TKA patients’ score (mean rank = 25.62) (U = 6.000, Z = −6.606, p ≤ 0.001). Thus, patients who required TKA had a significantly lower KSS score than patients who required other treatments.

Conclusion

According to the data obtained in the studied group of patients, the characteristics of the patient at high risk of requiring TKA are the following: a female patient over 65 years of age who associates hypertension, high ESR, and fibrinogen values ​​with KSS score and KSS function with low values, recording an average value of 56.70.

## Introduction

Knee osteoarthritis (KOA) is the most common form of osteoarthritis, whose diagnosis is based on clinical symptoms, physical examination, and imaging, most frequently by X-ray of the knee in at least two views [1]. KOA is characterized by joint swelling, pain, and gradual loss of function of the knee, with a significant impact on quality of life and daily activities [2]. With the increase in life expectancy and the need for an active life, diagnosis and treatment of this condition have become increasingly challenging, requiring a series of non-surgical therapeutic interventions, as well as surgical interventions in the advanced stages. Estimates show that approximately 10% of people over 50 years of age will be affected by osteoarthritis of the knee [3], with this percentage increasing with increased obesity, life expectancy, and associated pathologies.

The etiology of KOA, initially considered an inflammatory disease, remains not fully understood. The lack of therapeutic response to anti-inflammatory treatment and adjuvant therapies has led to the formulation of much more complex theories beyond the inflammatory mechanism. With the introduction of molecular diagnostics, understanding etiopathogenic mechanisms and modifying therapeutic interventions adapted to each patient have increased. KOA can be primary, without an identified cause, or secondary, in which a trigger is identified, and treatment can address the cause and not the symptoms. The main causes of secondary KOA are trauma, inflammatory autoimmune disease, joint instability, infection, neglected meniscal injury, ligament laxity, and patient activity level [4].

Although there is an important genetic component in primary KOA, the genes responsible for the condition and those with therapeutic potential are still under investigation [3]. Identifying the genes responsible for KOA is imperative for understanding its etiopathogenesis and identifying target genes that allow for disease-modifying treatment [4].

Diagnosis and initiation of surgical or conservative treatment are based on clinical and imaging examination [5]. The clinical examination considers the intensity of the pain, the degree of mobility of the knee, and the limitation of movements or stiffness. Calculating the functional scores at the time of the patient’s presentation to the orthopedic surgeon is essential, especially in the case of patients admitted for total knee arthroplasty (TKA), as it allows for the quantification of the post-operative result and improved quality of life. Such quantification is represented by the Knee Society Score (KSS), which has an objective component completed by the orthopedic surgeon considering the degree of mobility, pain, and stability of the knee, but also a subjective component that considers the patient’s complaints of walking distance and ability to climb stairs [4].

Indications of knee arthroplasty in the case of patients with KOA include the patient’s age (i.e., over 50 years), the presence of radiological changes of arthrosis, and the lack of response to conservative treatment after excluding other differential diagnoses. The persistence of pain and impairment of mobility in the case of middle-aged patients without clear radiological changes of osteoarthritis calls for the use of other diagnostic means with a higher degree of specificity and sensitivity. There is great difficulty in making therapeutic decisions in patients with early KOA and progressive worsening of symptoms over a very short time, with important consequences for daily activity and quality of life [6].

Magnetic resonance imaging (MRI) represents a more sensitive imaging method in terms of identifying cartilage lesions, in addition to other lesions that can generate pain [5]. However, the availability and related costs of MRI still make conventional radiography the gold standard in the diagnosis of KOA. KOA has a complex pathology, which, in addition to changes in bone structure and articular cartilage, involves the entire knee joint, generating changes in the menisci, ligaments, synovial, synovial fluid, and muscle structures [5]. The attitude toward a patient with early KOA is to wait and follow the radiological changes in the dynamics or to use investigations with higher sensitivity and specificity than conventional radiography [6].

Another promising direction in the diagnosis and quantification of KOA progression is represented by the quantitative determination of biomarkers in blood, urine, or synovial fluid. Biomarkers can be effector molecules directly involved in the destruction of articular cartilage or cartilage extracellular matrix fragments (e.g., hyaluronan), which serve as both biomarkers and stimuli of the innate immune chronic wound-healing response in the osteoarthritic joint [7,8]. Despite the vast research on the biomarkers involved in KOA, none has been established in current clinical practice as the gold standard in the diagnosis and treatment of this pathology [9,10].

Molecular diagnosis using gene sequencing techniques also shows potential in KOA diagnosis and treatment. Changes in gene expression occur at the level of articular cartilage, menisci, ligaments, synovial tissue, and synovial fluid. Identification of the genes and signaling pathways involved in the degradation of articular cartilage and other changes is an essential component in the identification of disease-modifying drugs [11].

In patients with early KOA, all predictive factors and risk factors that can be modified, as well as those that can lead to a rapid evolution of the symptoms and the need for TKA, must be considered and identified. In a systematic review, Belo et al. identified a series of prognostic factors associated with KOA, including age, gender, body mass index (BMI), degree of physical activity, decrease in bone mineral density, C-reactive protein (CRP), malalignment, clinical severity at baseline, and previous traumas [12].

Bastick et al., in a systematic review, identified significant associations between age, ethnicity, BMI, comorbidity count, MRI-detected infrapatellar synovitis, joint effusion, and baseline OA severity (radiographic and clinical) and KOA progression [13].

Treatment of KOA is varied and involves pharmacological and non-pharmacological measures and surgical treatment in the final stages of evolution. Surgical treatment of KOA is represented by TKA, which involves reconstruction of the knee joint. Post-operative results are predictable and lead to a high degree of patient satisfaction with improved mobility, decreased pain, and increased quality of life. TKA represents a very good solution for patients with damage to two or three compartments and those who do not see improvement after conservative treatment [14,15]. Furthermore, the need for correction of a significant or progressive deformity at the knee with evidence of osteoarthritis can also be an indication of a TKA.

TKA is a reliable surgical procedure with a predictable outcome in the appropriate patient, with reported survival rates of 85% over 10-25 years of follow-up [16,17]. Although TKA represents a surgical intervention with a high degree of patient satisfaction, it is also burdened by a series of risks and complications that can lead to an increase in mortality and morbidity, especially in the case of elderly patients or patients with multiple comorbidities. The identification of patients who may need TKA in the near future is absolutely necessary so as to allow for correct planning and good control of associated pathologies, such as hypertension or diabetes, in order to reduce the risks associated with this type of surgical intervention.

## Materials and methods

Patients with KOA presenting in the Department of Orthopedics and Traumatology of the University Emergency Hospital of Bucharest, Bucharest, Romania, who required surgical treatment, such as TKA, arthroscopy, or non-surgical treatment, were enrolled in this descriptive and prospective observational study. The study included 70 patients diagnosed with KOA either clinically or through imaging. The study was conducted between January 1, 2023, and March 31, 2024. The inclusion criteria consisted of patients over 45 years old with knee pain and radiological signs of KOA. The exclusion criteria consisted of patients with recent traumatic history, signs of active osteo-articular infection, lack of radiological changes of KOA that imposed a differential diagnosis, patients known to have rheumatological diseases in the Algic phase, and patients in whom it was not possible to collect complete required data. Study approval was obtained from the local ethics committee of the University Emergency Hospital of Bucharest, and informed consent was obtained from all participants.

Statistical analysis was performed using IBM SPSS Statistics, version 21.0 (IBM Corp., Armonk, NY). Data were collected using Excel 6.2.14 (v16.0). The collected data comprised age, gender, BMI, presence of diabetes, presence of hypertension, and values of inflammatory markers, such as erythrocyte sedimentation rate (ESR), CRP, and fibrinogen. KSS was also calculated at the presentation, and the radiological stage was established using the Kellgren and Lawrence classification.

The aim of this study was to identify the prognostic factors that predict the need for knee arthroplasty in patients with KOA by analyzing a series of risk factors known in the literature. In addition, we aimed to identify a biological pattern of patients requiring TKA by comparing those who benefited from TKA with a control group who only required pharmacological treatment or minimally invasive surgical procedures.

After obtaining informed consent, the patients underwent knee radiography (anterior and lateral views) to determine the degree of arthrosis according to the Kellgren and Lawrence classification. Blood samples were collected to obtain the necessary biological parameters. The patients were weighed, and their height was measured to obtain their BMI; the Oxford KSS was applied at the presentation.

Statistical tests were performed to assess associations between the abovementioned parameters and tests that identified risk factors, including odds ratios, the chi-squared test, the calculation of Cramer’s V and Phi coefficients, the Mann-Whitney U test, and other necessary tests. The normality of distributions was established using the Kolmogorov-Smirnov and Shapiro-Wilk tests and graphical analysis. Levene’s test was used to verify the homogeneity of the distributions. A p-value of <0.05 was considered statistically significant.

Finally, the participants were divided into TKA and non-TKA groups, with the latter representing patients who did not require TKA and who benefited from other alternative therapies, such as knee arthroscopy, anti-inflammatory treatment, physical therapy, injection of viscoelastic substance, and modifications of lifestyle.

## Results

The study sample comprised 70 patients: 50 (71.43%) received TKA, and 20 (28.57%) received adjuvant treatments that did not include TKA (Table 1).

**Table 1:** Patient demographics and baseline characteristics ESR, erythrocyte sedimentation rate; CRP, C-reactive protein; BMI, body mass index; KSS, Knee Society Score; TKA, total knee arthroplasty

	Age	Sex	ESR (mm/h), (N value = 5-10)	CRP (mg/dL), (N value = 0-0.5)	Fibrinogen (N value = 238-498)	Diabetes	High blood pressure	BMI (N value = 18.5-24.9)	KSS knee score	KSS function score	Treatment (TKA/non-TKA)
1	67	F	5	2	359	No	Yes	34	60	60	TKA
2	68	F	9	3	320	No	Yes	31	50	42	TKA
3	81	F	27	1.8	425	Yes	Yes	30.2	50	50	TKA
4	64	M	18	0.74	377	No	Yes	34.2	60	50	TKA
5	51	F	4	0.03	382	No	Yes	36.7	60	50	TKA
6	62	F	17	0.02	424	No	Yes	35.8	70	60	TKA
7	67	F	3	1.34	342	Yes	Yes	33.6	70	60	TKA
8	67	F	12	0.3	326	No	Yes	34.9	35	24	TKA
9	54	F	11	0.3	387	No	Yes	33.8	70	60	TKA
10	68	F	7	0.82	312	Yes	No	26.4	70	60	TKA
11	65	M	12	3.22	354	No	Yes	30.9	60	50	TKA
12	68	F	8	0.4	325	No	Yes	28.3	35	35	TKA
13	74	M	26	7	434	No	Yes	22.5	60	50	TKA
14	69	F	12	0.4	390	No	Yes	33.8	60	60	TKA
15	65	F	12	0.66	348	No	Yes	27	60	50	TKA
16	56	F	33	0.5	385	No	Yes	34.3	70	60	TKA
17	64	F	17	2.17	417	Yes	Yes	30.9	60	50	TKA
18	67	F	16	1.2	366	No	Yes	33.2	60	60	TKA
19	55	M	3	0.64	292	No	Yes	28.1	60	50	TKA
20	77	F	10	0.12	282	Yes	Yes	30.1	35	24	TKA
21	69	M	13	0.58	388	No	Yes	31	60	50	TKA
22	75	F	8	0.4	341	Yes	Yes	29.2	60	35	TKA
23	62	F	27	0.4	333	No	Yes	34.5	60	35	TKA
24	74	F	10	0.23	289	No	Yes	32	35	24	TKA
25	59	M	11	0.3	294	No	Yes	26.1	70	60	TKA
26	65	M	5	0.95	280	No	No	32	60	50	TKA
27	62	F	14	0.64	319	Yes	Yes	31.4	35	24	TKA
28	72	F	9	0.25	325	No	Yes	30.2	60	50	TKA
29	73	F	11	0.68	368	No	Yes	29.1	60	42	TKA
30	71	M	12	0.5	302	No	Yes	29.2	35	35	TKA
31	44	F	6	0.4	326	Yes	No	31.2	35	35	TKA
32	76	F	11	1.32	279	No	Yes	31.4	40	24	TKA
33	76	F	10	0.99	407	No	Yes	30	60	50	TKA
34	55	M	8	0.8	409	No	Yes	29.2	70	50	TKA
35	58	F	13	9.41	394	No	Yes	28.1	60	42	TKA
36	71	M	18	1.5	420	No	Yes	30.1	60	50	TKA
37	75	F	26	2.3	379	Yes	Yes	33.2	35	35	TKA
38	56	F	11	0.99	384	No	No	29.8	60	50	TKA
39	62	M	6	0.5	312	No	No	27.2	60	50	TKA
40	72	F	11	0.4	337	No	Yes	30.2	35	35	TKA
41	45	F	8	0.5	436	No	Yes	31	70	50	TKA
42	69	F	11	0.8	370	No	Yes	29.2	60	50	TKA
43	64	F	8	1.02	375	No	Yes	27.5	70	42	TKA
44	69	F	9	0.87	265	No	Yes	29.7	60	50	TKA
45	59	F	13	1.04	317	No	Yes	30.4	70	50	TKA
46	63	F	5	0.62	329	No	No	31	70	50	TKA
47	65	F	10	0.5	377	No	Yes	29.8	70	60	TKA
48	74	F	60	5.93	396	No	Yes	33.5	50	24	TKA
49	59	M	23	6.17	474	Yes	Yes	33	50	42	TKA
50	53	F	21	1.5	395	Yes	Yes	29.8	60	50	TKA
51	51	F	3	2	367	No	Yes	38	80	80	Non-TKA
52	56	M	3	0.02	254	Yes	Yes	33.2	90	90	Non-TKA
53	45	F	40	0.05	322	No	Yes	27	90	90	Non-TKA
54	55	F	5	0.6	345	No	No	27.2	80	90	Non-TKA
55	47	F	4	0.4	350	No	No	28.1	80	80	Non-TKA
56	59	M	6	0.8	340	No	No	30.5	70	80	Non-TKA
57	55	M	5	1	491	No	No	32.1	80	80	Non-TKA
58	55	M	7	0.7	296	Yes	Yes	32.2	80	70	Non-TKA
59	53	M	4	0.9	299	No	Yes	33.2	80	80	Non-TKA
60	49	M	5	1	312	No	Yes	31.2	90	80	Non-TKA
61	52	F	6	0.7	287	No	Yes	29.8	90	80	Non-TKA
62	54	M	4	0.9	498	No	Yes	30.2	80	80	Non-TKA
63	51	F	5	0.7	344	No	No	28	80	90	Non-TKA
64	48	F	4	0.6	349	No	No	28.5	80	80	Non-TKA
65	58	M	5	1	352	No	No	30.1	90	80	Non-TKA
66	54	M	6	0.9	299	Yes	Yes	30.2	90	80	Non-TKA
67	55	F	8	1.4	302	No	Yes	29.7	80	90	Non-TKA
68	49	F	7	0.8	300	No	Yes	29.9	90	80	Non-TKA
69	53	M	6	1.1	305	Yes	Yes	31.2	80	80	Non-TKA
70	52	M	5	1.2	288	Yes	No	29.8	80	90	Non-TKA

A t-test was performed to determine whether there were age differences between patients who did not have TKA and those who did have TKA. There were no extreme values according to the graphical analysis. The ages of the patients in the two groups were normally distributed according to the Shapiro-Wilk test (p > 0.05), and the variances were not homogeneous according to the Levene test for equality of variances (p = 0.003). Patients who required TKA were older (mean age = 65.12 years, SD = 8.19, CI [62.79, 67.45]) than those who required other therapeutic interventions (mean age = 52.55 years, SD = 3.63, CI [50.85, 54.25]), with the difference of 12.57 years being statistically significant (t = −8.882, p < 0.05) (Table 2).

**Table 2:** The incidence of the main predictive factors in the patient group Data are represented as %. ESR, erythrocyte sedimentation rate; CRP, C-reactive protein; TKA, total knee arthroplasty

Variables	TKA group	Non-TKA group
Mean age (years)	65.12	52.55
Female	76%	45%
Male	24%	55%
Diabetes (yes)	22%	25%
High blood pressure (yes)	88%	60%
ESR value (high)	58%	5%
CRP value (high)	72%	85%

The chi-squared test indicated that there were statistically significant differences between the two groups of patients in terms of gender (Χ^2^ = 6.223, p = 0.013), and Cramer’s V and Phi coefficients (0.298) revealed that the link between the two variables was direct but weak in intensity (p = 0.013). These statistical tests indicated that the female gender increased the risk of requiring TKA.

An OR was calculated to assess the association between patient gender and TKA. Women were more than three times more likely to require TKA than men (80.85% vs. 52.17%, OR = 3.87, CI [1.29; 11.56]) (Table 3).

**Table 3:** Distribution of cases in the two groups according to the main predictive factors Data are represented as %. ESR, erythrocyte sedimentation rate; TKA, total knee arthroplasty

Variables	TKA group	Non-TKA group
Female	80.85%	19.15%
Male	52.17%	47.83%
High blood pressure (yes)	78.57%	21.43%
High blood pressure (no)	42.86%	57.14%
ESR value (normal)	52.50%	47.50%
ESR value (high)	96.67%	3.33%

Diabetes was not identified as a predictive factor or with an increased incidence in the case of patients who benefited from TKA, with no statistically significant difference found in the study sample (p > 0.05) (Table 2).

Hypertension was frequently encountered in the study sample. The chi-squared test indicated a statistically significant association between the presence of high blood pressure and the type of treatment required by the patient (Χ^2^ = 7.000, p = 0.008). Cramer’s V and Phi coefficients (0.316) revealed that the association between the two variables was direct and moderate in intensity (p = 0.008). This statistical test indicated that patients with high blood pressure had a moderate tendency to require TKA over other treatments (Table 3).

An OR was calculated to assess the association between the presence of hypertension and TKA. Patients with hypertension were more than four times more likely to require TKA than patients without hypertension (78.57% vs. 42.86%, OR = 4.88, CI [1.42, 16.82]) (Table 3).

The chi-squared test indicated that there was a statistically significant association between the ESR value and the type of treatment required by the patient (Χ^2^ = 16.386, p ≤ 0.001). Cramer’s V and Phi coefficients (0.484) revealed that the association between the two variables was direct and moderate in intensity (p ≤ 0.001). This statistical test, together with the graphical representation, indicated that among patients who required TKA, there was a moderate tendency to have elevated ESR levels compared to patients who did not require TKA, with the latter tending to have ESR values within normal limits.

An OR was calculated to assess the association between elevated ESR and TKA. Patients with elevated ESR were more than 26 times more likely to require TKA than patients with normal ESR (96.67% vs. 52.50%, OR = 26.23, CI [3.25; 211.67]) (Table 3).

The TKA group had CRP levels ranging from 0.03 to 9.41 mg/dL, with a mean value of 1.38 mg/dL (SD = 1.88, CI [0.85, 1.92]) and a median value of 0.71 mg/dL. 

Both groups exhibited above-normal/increased values of CRP, with non-TKA patients presenting such values more frequently. On the other hand, among patients with TKA, several cases with extreme values (outliers) of CRP were found. No statistically significant differences were noted between the two compared groups in terms of CRP (p > 0.001) (Table 2).

**Figure 1:**
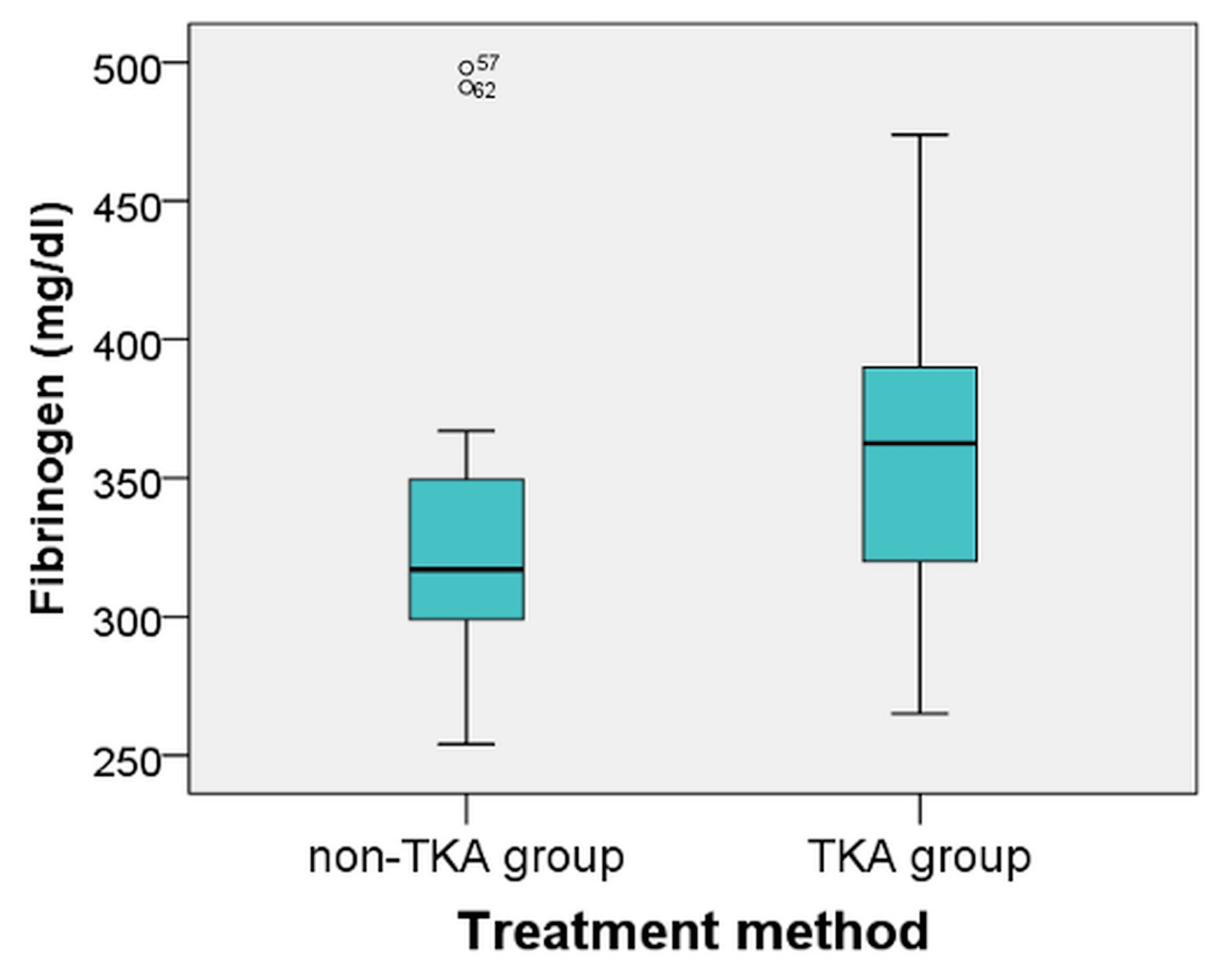
Distribution of cases according to fibrinogen level Data are represented as N. TKA, total knee arthroplasty

The Mann-Whitney U test was applied to determine whether there were differences between groups regarding fibrinogen level. The distribution of fibrinogen levels varied between the two groups (Figure 1). There was a statistically significant difference between the fibrinogen levels of the non-TKA group (mean rank = 27.13) and that of the TKA group (mean rank = 38.85) (U = 332.500, Z = −2.178, p = 0.029). Therefore, although all patients presented fibrinogen within normal limits, those who required TKA had significantly higher values than those who did not require TKA.

Patients who received treatments other than TKA had a KSS between 70 and 90, with a mean value of 83 (SD = 5.71, CI [80.33, 85.67]) and a median value of 80. In the TKA group, KSS ranged from 35 to 70, with a mean value of 56.70 (SD = 12.02, CI [53.28, 60.12]) and a median value of 60.

**Figure 2:**
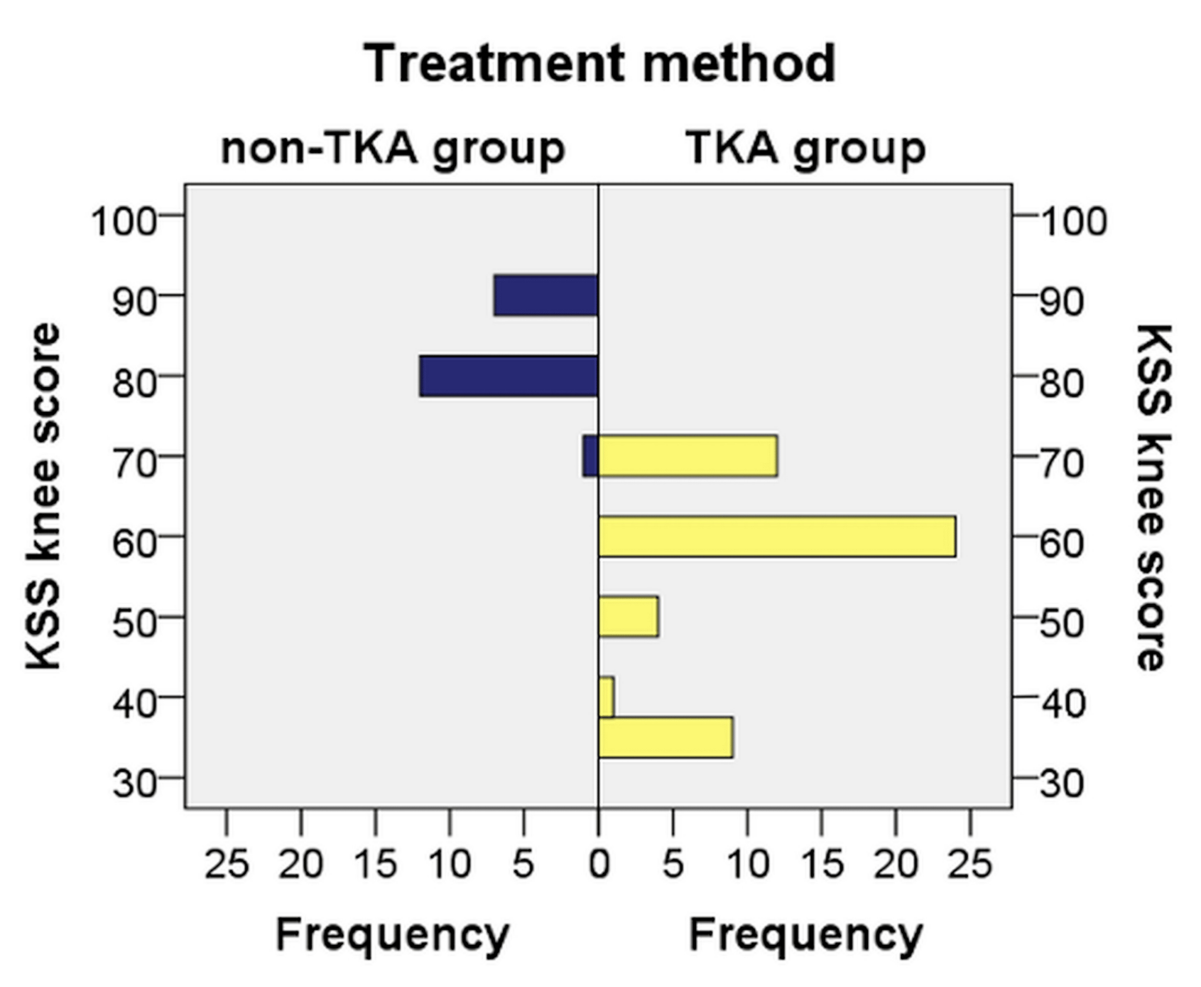
Distribution of cases according to KSS Data are represented as N. KSS, Knee Society Score; TKA, total knee arthroplasty

The distribution of KSS values in the two analyzed groups was not similar, as can be seen from the graphical analysis. There was a statistically significant difference between the non-TKA group (mean rank = 60.20) and the TKA group (mean rank = 25.62) (U = 6.000, Z = −6.606, p ≤ 0.001). Therefore, patients who required TKA had a significantly lower KSS than patients who required other treatments (Figure 2).

**Figure 3:**
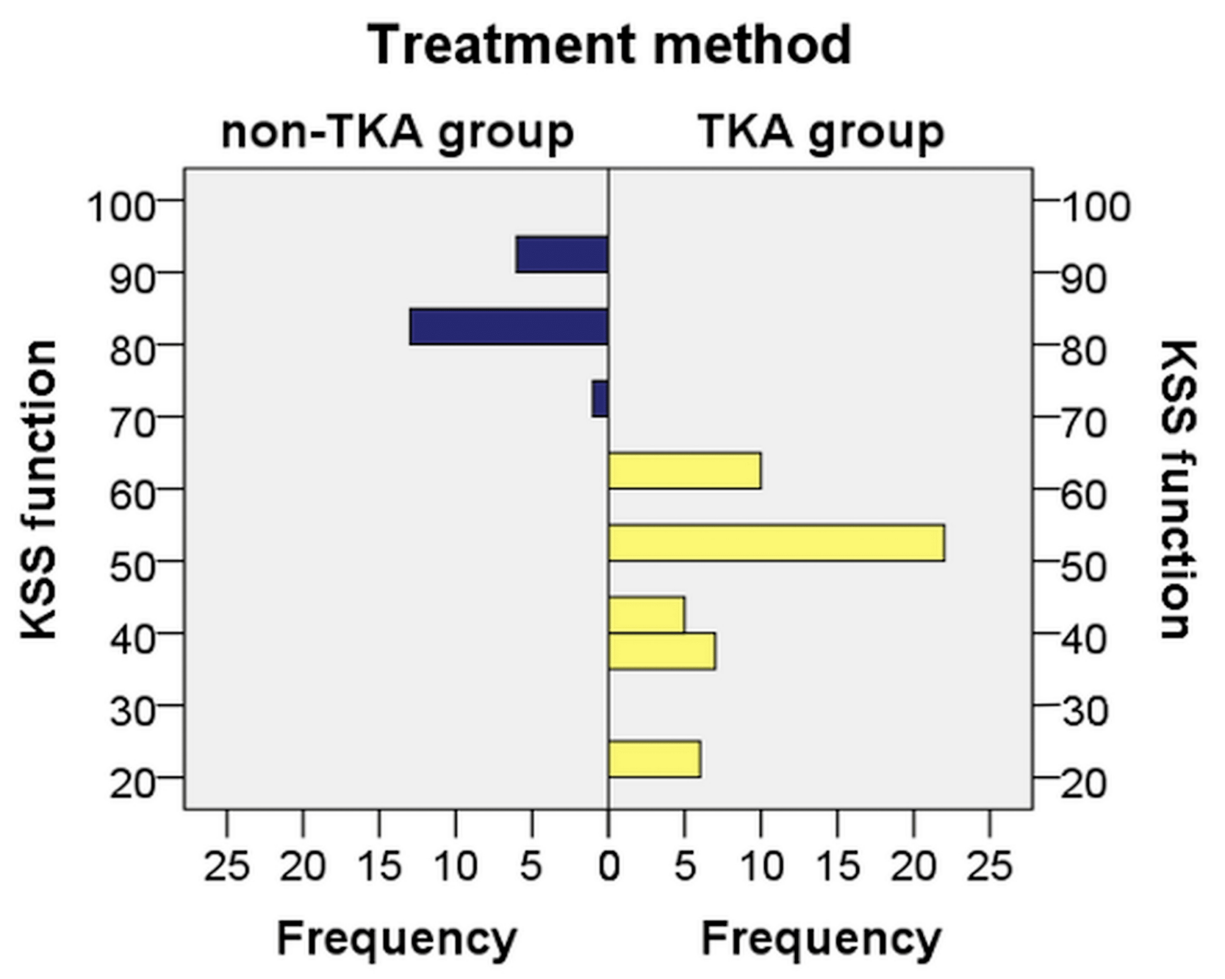
Distribution of cases according to KSS function score Data are represented as N. KSS, Knee Society Score; TKA, total knee arthroplasty

Regarding the KSS function score, the distribution of score values in the two analyzed groups was different. There was a statistically significant difference between the KSS function score of patients in the non-TKA group (mean rank = 60.50) and that of patients in the TKA group (mean rank = 25.50) (U = 0.000, Z = −6.644, p ≤ 0.001). Therefore, patients who required TKA had significantly lower functional KSS values than patients who required other treatments (Figure 3).

## Discussion

Among our sample, some of our results agreed with those of pre-existing studies, whereas others may lead to the opening of new research themes on large groups of patients. Advanced age (i.e., people over 65 years old) represents a risk factor for the need for TKA [18,19]. Female gender was also a risk factor for the need for TKA, with women presenting a three times higher risk of requiring TKA than men, an aspect also observed by Collins et al. in a profile study that included 1753 participants [20]. Although some studies have cited diabetes as a risk factor in the evolution of KOA, it was not identified as a predictive factor for the need for TKA in our sample studied at the University Emergency Hospital of Bucharest, as compared to hypertension, which had a direct association with the need for TKA. Blood pressure values can also be modified by the administration of pain treatment, sometimes in excess. All enrolled patients had chronic antihypertensive treatment at the time of admission, and we cannot specify how much blood pressure values were influenced by the previous KOA treatment.

The association of diabetes with hypertension and obesity leads to the establishment of metabolic syndrome, which is cited in some studies as having a role in the progression of KOA. For example, Eymard et al., in a study that included 559 patients with KOA, demonstrated that type 2 diabetes was associated with the progression of the arthrosis, but in patients who were already diagnosed with this pathology [21].

The inflammatory markers in the studied group had a heterogeneous distribution, so ESR values were increased in those who required TKA, correlating positively with this. In contrast, CRP did not show a positive association with TKA, but extreme values were identified in the TKA group. Changes in inflammatory markers are associated with KOA, with high values of ESR and CRP associated with KOA evolution and painful symptomatology [22]. At the same time, the extreme values of CRP explain the KSS functional score and its identification in the TKA group.

Although fibrinogen levels fell within the normal range for both groups in this study, they were greatly increased in the TKA group compared to the non-TKA group. A laboratory study conducted by Flick et al. identified fibrinogen as being involved in the pathogenesis of KOA [22]. Thus, fibrinogen is an important but context-dependent determinant of arthritis, with alphaMbeta2-mediated inflammatory processes being one potential mechanism linking fibrinogen to joint disease. Regarding the values of KSS and KSS function score, the results were predictable and in accordance with our expectations and data from the literature, with patients who required TKA presenting much lower values [23].

The identification of the predictive factors of the evolution of KOA is crucial, both in adapting treatment to the needs of patients and from an economic point of view. Increased life expectancy and the need for an active lifestyle lead to an increased need for TKA, which leads to an increased economic burden on the national health system. The predictive factors identified in this study are not necessarily modifiable but can be used to identify patients at risk and to monitor the evolution of the disease. There were no significant associations regarding modifiable factors (e.g., BMI) except hypertension, which had a direct association with the risk of TKA.

The study's limitations are represented by the relatively small number of patients and the lack of follow-up in terms of the postoperative functional score and the values of the identified predictive factors. It should also be mentioned that it is a study carried out in a single center, and the need to perform multicenter studies is a real one with well-known advantages.

## Conclusions

The predictive factors that showed significant associations with the risk of requiring TKA included the patient’s age and female gender, with women having three times higher risk of TKA compared to men. In addition, patients with hypertension, together with elevated ESR and fibrinogen values, showed statistically significant direct associations with the need for TKA. Although within normal parameters, fibrinogen values showed increased values in patients who required TKA, which opens the opportunity for new molecular biology research directions through its association with alphaMbeta2-mediated inflammatory processes. According to the data obtained in the study sample, patients with a high risk of requiring TKA were characterized as follows: female patients over 65 years of age with associated hypertension, high ESR and fibrinogen values, and low KSS and KSS function, recording an average value of 56.70.
